# Temporal Beta Diversity of Bird Assemblages in Agricultural Landscapes: Land Cover Change vs. Stochastic Processes

**DOI:** 10.1371/journal.pone.0127913

**Published:** 2015-05-26

**Authors:** Andrés Baselga, Sébastien Bonthoux, Gérard Balent

**Affiliations:** 1 Departamento de Zoología, Facultad de Biología, Universidad de Santiago de Compostela, Rúa Lope Gómez de Marzoa s/n, 15782 Santiago de Compostela, Spain; 2 INRA, UMR 1201 DYNAFOR, F-31320 Castanet-Tolosan, France; 3 ENSNP, UMR 7324 CITERES, 9 rue de la chocolaterie, 41000 Blois, France; Universidad de Granada, SPAIN

## Abstract

Temporal variation in the composition of species assemblages could be the result of deterministic processes driven by environmental change and/or stochastic processes of colonization and local extinction. Here, we analyzed the relative roles of deterministic and stochastic processes on bird assemblages in an agricultural landscape of southwestern France. We first assessed the impact of land cover change that occurred between 1982 and 2007 on (i) the species composition (presence/absence) of bird assemblages and (ii) the spatial pattern of taxonomic beta diversity. We also compared the observed temporal change of bird assemblages with a null model accounting for the effect of stochastic dynamics on temporal beta diversity. Temporal assemblage dissimilarity was partitioned into two separate components, accounting for the replacement of species (i.e. turnover) and for the nested species losses (or gains) from one time to the other (i.e. nestedness-resultant dissimilarity), respectively. Neither the turnover nor the nestedness-resultant components of temporal variation were accurately explained by any of the measured variables accounting for land cover change (r^2^<0.06 in all cases). Additionally, the amount of spatial assemblage heterogeneity in the region did not significantly change between 1982 and 2007, and site-specific observed temporal dissimilarities were larger than null expectations in only 1% of sites for temporal turnover and 13% of sites for nestedness-resultant dissimilarity. Taken together, our results suggest that land cover change in this agricultural landscape had little impact on temporal beta diversity of bird assemblages. Although other unmeasured deterministic process could be driving the observed patterns, it is also possible that the observed changes in presence/absence species composition of local bird assemblages might be the consequence of stochastic processes in which species populations appeared and disappeared from specific localities in a random-like way. Our results might be case-specific, but if stochastic dynamics are generally dominant, the ability of correlative and mechanistic models to predict land cover change effects on species composition would be compromised.

## Introduction

Understanding the impacts of global change on species assemblages is of crucial importance for biodiversity conservation. Across the globe, local assemblages have been shown to be the subject of marked temporal variation, beyond that predicted from null models, which suggests deterministic effects of environmental change [1]. The impact of deterministic processes such as climate or land cover change on biological communities has been documented for multiple taxa and geographical regions [2–4]. However, one of the most critical problems is how to discern the effect of these deterministic processes from the natural baseline turnover of species assemblages [5] or the effect of stochastic processes [6]. This problem is analogous to the one in spatial analyses aiming to separate the effects of niche processes and neutral dispersal limitation in driving assemblage dissimilarity across space [7–9].

At local to regional scales, land cover change is one of the most relevant deterministic processes controlling the dynamics of species distributions [10, 11]. Undisputedly, the habitat requirements of species are a key determinant of distribution shifts when human activities produce changes in the distribution of habitats through land cover change. For example, in Mediterranean regions the forest maturation and spread due to land abandonment has induced a shift of bird communities in favour of woodland species [12, 13]. Additionally, agricultural intensification and a reduction in low intensity managed lands has lead to a decline in farmland bird species [14, 15]. At the assemblage level, the temporal changes in assemblage composition (i.e. temporal beta diversity) would be the result of these deterministic changes in species distributions and, therefore, would be related to the temporal changes in land cover. However, besides (or perhaps beneath) deterministic effects controlled by environmental changes through niche filtering, stochastic occupancy dynamics could also be responsible for changes in the local distribution of species. Local random extinctions and dispersal events from occupied to non-occupied suitable habitats would produce neutral shifts in species distributions that, at the assemblage level, would result in neutral variation in assemblage composition through time [16].

Because both types of process (deterministic and neutral) may be operating simultaneously (i.e. forming a continuum from purely deterministic to purely neutral), it is necessary to test their relative contributions, which are likely to vary between biological systems. In this paper, we assess temporal taxonomic beta diversity in bird assemblages in southwestern France. The assessment of temporal changes in species assemblages can be conducted at the alpha and beta diversity levels. At the alpha diversity level, different diversity indices (i.e. species richness, Shannon entropy…) can be used to quantify changes in local diversity of assemblages from one time to another [17, 18]. At the beta diversity level, temporal changes in community composition can be quantified using dissimilarity indices [2, 6, 19]. Both approaches are complementary as they assess different facets of biodiversity. In the case of temporal beta diversity, it is important to stress that temporal changes in species composition can be related to both (i) the replacement of species from time to time (temporal turnover) and (ii) the gain or loss of species forming nested subsets from time to time (nestedness-resultant dissimilarity) [20, 21]. Both phenomena, replacement and species loss/gain, could be either related to stochastic or deterministic processes, but the relationships between turnover or nestedness-resultant patterns and predictors could be different, or even collapse each other (see [22] for an example involving spatial patterns). Therefore, the antithetic turnover and nestedness-resultant components of temporal beta diversity have to be separated to assess the respective influence of deterministic and stochastic processes on them. Furthermore, the spatial pattern of beta diversity (again both the replacement and nestedness components) can also be subject of temporal changes, which can also be linked to deterministic processes (for example, if rare species are consistently removed, biotic homogenization reduces spatial beta diversity) or to stochastic processes. Significant temporal changes in multiple-site dissimilarity across the region, and directional (not random) changes in the specific structure of spatial pairwise dissimilarities would point to the effect of deterministic processes. Under stochastic dynamics, in contrast, the overall biotic heterogeneity is expected to remain unchanged, and the specific structure of spatial pairwise dissimilarities is expected to change randomly. In our study system, temporal changes in alpha diversity (species richness) have already been shown to be correlated with changes in land cover, although the observed temporal patterns were less consistent than their spatial counterparts [18]. Therefore, we here focus on temporal changes in species composition and spatial beta diversity, trying to discern the relative importance of stochastic and deterministic processed linked to land cover change across a period of 25 years. Specifically, our aims are to (i) quantify the correlation between temporal bird assemblage dissimilarity and amount of land cover change, considering that the response of assemblages might be different for increment than for decline in land covers’ surface, (ii) quantify the temporal changes in spatial beta diversity, and (iii) assess whether the observed changes in bird assemblage composition are compatible with neutral assemblage variation.

## Material and Methods

### Study site

The study site was the 'Coteaux de Gascogne', located in the Pyrenean Piedmont in south-western France. The study was carried out exclusively on private land, after agreement between owners and authorities to allow INRA researchers to perform long term investigations. For future sampling permits, researchers need to contact the head of the INRA UMR 1201 DYNAFOR lab (Dr. Marc Deconchat, marc.deconchat@toulouse.inra.fr). All the studied sites belong to the 'European Long-Term Ecological Research' network (LTER_EU_FR_003, "Valleys and Hills of Gascony"). This hilly region is temperate with frequent summer droughts. Annual precipitation is moderate (700 mm/year) and variable over the year with most rain falling in spring. Due to soils, topography and local agricultural traditions, landscape is heterogeneous with a combination of small woodlands (15% of the study site), hedgerows, fallow lands, permanent grasslands, and crops mainly including barley, wheat, maize silage, colza and sunflowers.

### Biological data and environmental predictors

We used 256 point counts (sites from here on) censused in 1982 with a stratified design representing the diversity of land-cover types (see [18] for more details on point counts distribution). These sites were again censused in 2007. Bird communities were recorded during 20 minutes in a 125 m radius, representing an area of about 5 ha. This area is larger than the home range of most of the studied species [23]. Sampling consisted in recording species presences by sight and/or song, without manipulation of specimens. Sites were censused once a year and during the breeding period (May). Sampling was restricted to days without marked rainfall or wind and during the period of vocal activity (4 h after sunrise). Raptors were excluded from analyses as the point count method is not suited to their large home ranges. Human-associated species were excluded because they are distributed close to human housings. In total, 47 species were considered. None of these species is endangered, although most of the species are protected by law. The species number per site ranged from 0 to 18 and averaged 7.1 (SD = 3.2).

**Fig 1:**
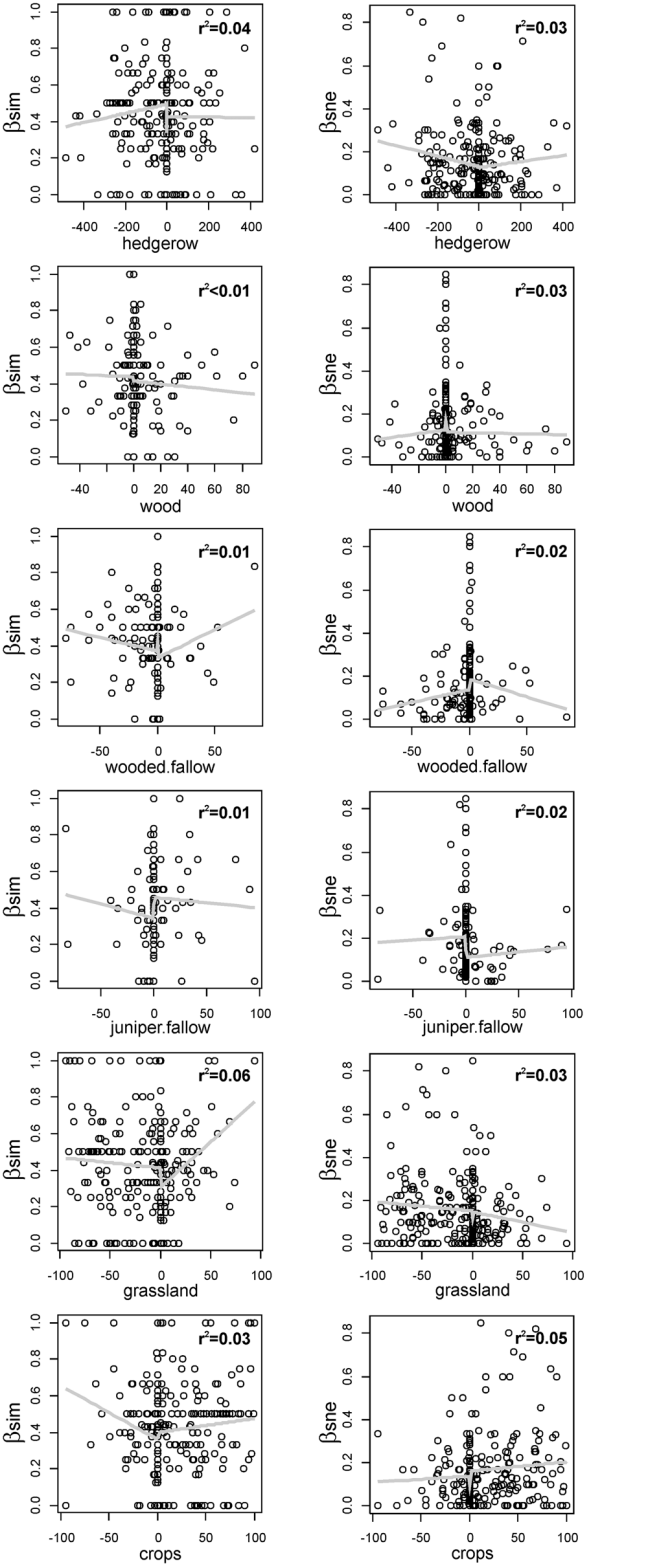
Relationship between temporal dissimilarity (turnover and nestedness-resultant components in the left and right column, respectively) and land cover change (by rows: temporal variation in hedgerow length, percentage of woodland area, wooded fallow area, juniper fallow area, permanent grassland area, crop area). Gray lines are the fitted functions from which r^2^ values were computed.

We quantified land cover within a 125m radius around each point count by using aerial photograph from 1979 for the 1982 census and the BDOrtho orthorectified digital photograph database (French National Geographical Institute, IGN) from 2006 for the 2007 census. The interpretation of photographs was checked in the field during bird censuses. Six land covers relevant for explaining bird distributions [24] were digitised in the sampling area: percentage of woodland, wooded fallow, juniper fallow, permanent grassland, crop, and the total length of hedge. Woodlands are dominated by *Quercus robur* L. and *Quercus pubescens* Willd., present alongside *Carpinus betulus* L., *Prunus avium* L. and *Sorbus torminalis* (L.) Crantz. Wooded fallow grows after wood cutting. This habitat is densely covered with shrubs and trees. Juniper fallow (*Juniperus communis* L.) grows on undergrazed grasslands on steep slopes. Permanent grasslands are not reseeded for at least five years. Crops essentially include cereals as wheat, barley and maize, oil crops as sunflower and colza, and forage crops as grasses and legumes. Other land covers such as housed areas and small roads were not included due to their low representation in the landscape. Between 1982 and 2007, there was a large increase in crop area (from an average of 27% in 1982 to 45% in 2007) at the expense of permanent grassland (from 33% to 17%). Average changes in the amount of woodland (from 25% to 27%) and the length of hedgerow (from 169 m to 142 m) were lower, but large changes (i.e. up to an increase of 89% or a decrease of 51% in woodland area) were still observed in some sites (Fig 1). The average absolute change in the dominant land cover class was 32% (SD = 27), with 26% of sites suffering an absolute change larger than 50%, and 68% of sites suffering an absolute change larger than 10%. Biological and land cover data are provided as *csv* files in Supporting Information (S1–S5 Datasets).

### Assessing temporal beta diversity and its correlates

The variation in assemblage species composition across time was measured as the dissimilarity between the point assemblages in 1982 and 2007 using the command *beta*.*temp* in package *betapart* [25] for R [26]. This procedure computes the overall dissimilarity (here measured as Sørensen dissimilarity, β_sor_) between the two times, and partitions it into its turnover (β_sim_) and nestedness-resultant (β_sne_) components [20, 21]. In the context of temporal variation of communities these two components reflect (i) the substitution of some species by others through time (β_sim_), and (ii) the loss (or gain) of species through time in a nested pattern (i.e. the assemblage at one time is a subset of the one observed at the other time, β_sne_), respectively. To avoid undefined values in dissimilarities caused by null denominators, we removed all localities where no species were detected either in 1982 or 2007, so 252 localities were included in all subsequent analyses. Given that we had 252 localities at two times (1982 and 2007), we got 252 dissimilarity values per each dissimilarity component (β_sim_ and β_sne_). To assess the contribution of deterministic processes, the relationship between the variation in community composition and land cover change was estimated using a linear regression between dissimilarity (β_sim_ and β_sne_) and the variation in each land cover variable [e.g., Δwood = wood2007—wood1982]. Because the response of assemblages is expected to differ for positive (e.g., increment in wood area) and for negative (e.g., reduction in wood area) land cover changes, we performed piecewise regressions [27] with the breakpoint at zero value (i.e. no land cover change), using the *lm* function in R [26]. This procedure fits two different regression lines for positive and negative land cover changes, respectively. Given that the effect of absolute land cover change could differ for different levels of land cover area (i.e. a reduction from 20% to 10% in wood areas could be more relevant than a reduction from 70 to 60%) we repeated the piecewise regressions using a standardized measure of land cover change [e.g., std.wood = (wood2007—wood1982) / (wood2007 + wood1982+1). The unity added to the denominator was necessary to avoid undefined values.

### Assessing temporal change in spatial beta diversity

Besides temporal changes in assemblage composition (temporal beta diversity), the patterns of spatial beta diversity can also change with time. A potential effect of land cover change is the change in assemblage heterogeneity (for example, biotic homogenization). To assess this, we measured multiple-site spatial dissimilarity in 1982 and in 2007 for β_SIM_ and β_SNE_. To quantify the significance of the difference between multiple-site dissimilarity in 1982 and multiple-site dissimilarity in 2007, we computed 1000 multiple site dissimilarity values for 1982 and for 2007 by randomly sampling 50 sites, using the command *beta*.*sample* in R package *betapart* [25]. This created a distribution of multiple site dissimilarity for 1982 and another distribution for 2007, which were empirically compared (i.e. how many times would the opposite result be observed) to compute the significance of the difference between multiple site dissimilarity of 1982 and 2007. Another potential effect of land cover change could be a change in the particular dissimilarities between pairs of sites (i.e. the multivariate structure of pairwise spatial dissimilarities), which could make that pairs of sites being similar in 1982 would be dissimilar in 2007, and vice versa. To assess this, we performed two Mantel tests (i.e. one for β_sim_ and one for β_sne_) between 1982 and 2007 pairwise dissimilarity matrices using the *mantel* command in R package *vegan* [28]. Changes in the multivariate spatial structure of assemblages should be detected as low correlations between 1982 and 2007 dissimilarity matrices. This test informs about the changes in pair-wise spatial dissimilarity patterns, while the previous multiple-site test informs about changes in regional heterogeneity. Both analyses are complementary, as for example, two sites with identical composition in 1982 could become completely dissimilar in 2007, while two sites completely dissimilar in 1982 could become identical in 2007. This would change the multivariate pair-wise structure but not the overall heterogeneity.

### Assessing stochastic dynamics of assemblage variation

Temporal change in assemblages could be derived from stochastic population extinction and colonization events. To assess if the observed assemblage variation was compatible with stochastic dynamics, we first compared the empirical temporal dissimilarities with null dissimilarities intended to recreate stochastic changes in species composition. To do this null temporal changes were simulated by re-shuffling the species presences in localities while maintaining species frequencies constant (equivalent to null model FE in [29]). This simulates a regional bird meta-community where species randomly go extinct or colonize the specific localities from a common regional pool. Therefore, the reshuffling produces a null assemblage composition for each site (null model assemblages). The dissimilarity between 1982 assemblages and the null model assemblage (null model dissimilarities) was measured as for the empirical temporal change. We first compared, using Kolmogorov-Smirnov test, the observed distribution of temporal dissimilarities between the 1982 and 2007 assemblages with the distribution of null model dissimilarities. This test was repeated 1000 times using 1000 different null assemblages to assess the robustness of results. We then used these 1000 null models to compare the observed temporal dissimilarity of each site with the null distribution of temporal dissimilarities for that specific site. The observed temporal dissimilarity between 1982 and 2007 assemblages for each locality was compared with this distribution to detect those sites where temporal dissimilarity was significantly different from the null expectation. The test was two-sided, so we considered temporal dissimilarity significantly lower than null expectation if it fell below the 2.5 percentile of the null distribution, and significantly higher if it fell above the 97.5 percentile. Finally, we used ANOVA tests to assess whether sites with temporal dissimilarity significantly different from null distributions presented different intensity in their land cover change.

## Results

The temporal variation of site assemblages between 1982 and 2007 was dominated by species turnover (β_sim_) (mean = 0.42, SD = 0.25), implying that in any given site an average of 40% of the species were unique to the time with lower species richness (either 1982 or 2007 site assemblage), i.e. were not present at the time with higher species richness. In contrast, the nestedness-resultant component (β_sne_) was much lower (mean = 0.15, SD = 0.16), implying that no strong patterns of species losses or gains have occurred between 1982 and 2007. Neither the turnover (β_sim_) and nestedness-resultant (β_sne_) components of temporal beta diversity were significantly related to any of the measured variables accounting for land cover change (Fig 1, all r^2^<0.04, p>0.05), with the exception of the relationships between β_sim_ and hedgerow (r^2^ = 0.04, F_4,247_ = 2.75, p = 0.029) and grassland area (r^2^ = 0.06, F_4,247_ = 3.71, p = 0.006), and between β_sne_ and crops area (r^2^ = 0.05, F_4,247_ = 3.42, p = 0.010). If p values were Bonferroni corrected, all relationships would have turned to be not significant (i.e. all p>0.07). Similar results were observed when land cover change was standardized (S1 Fig).

**Fig 2:**
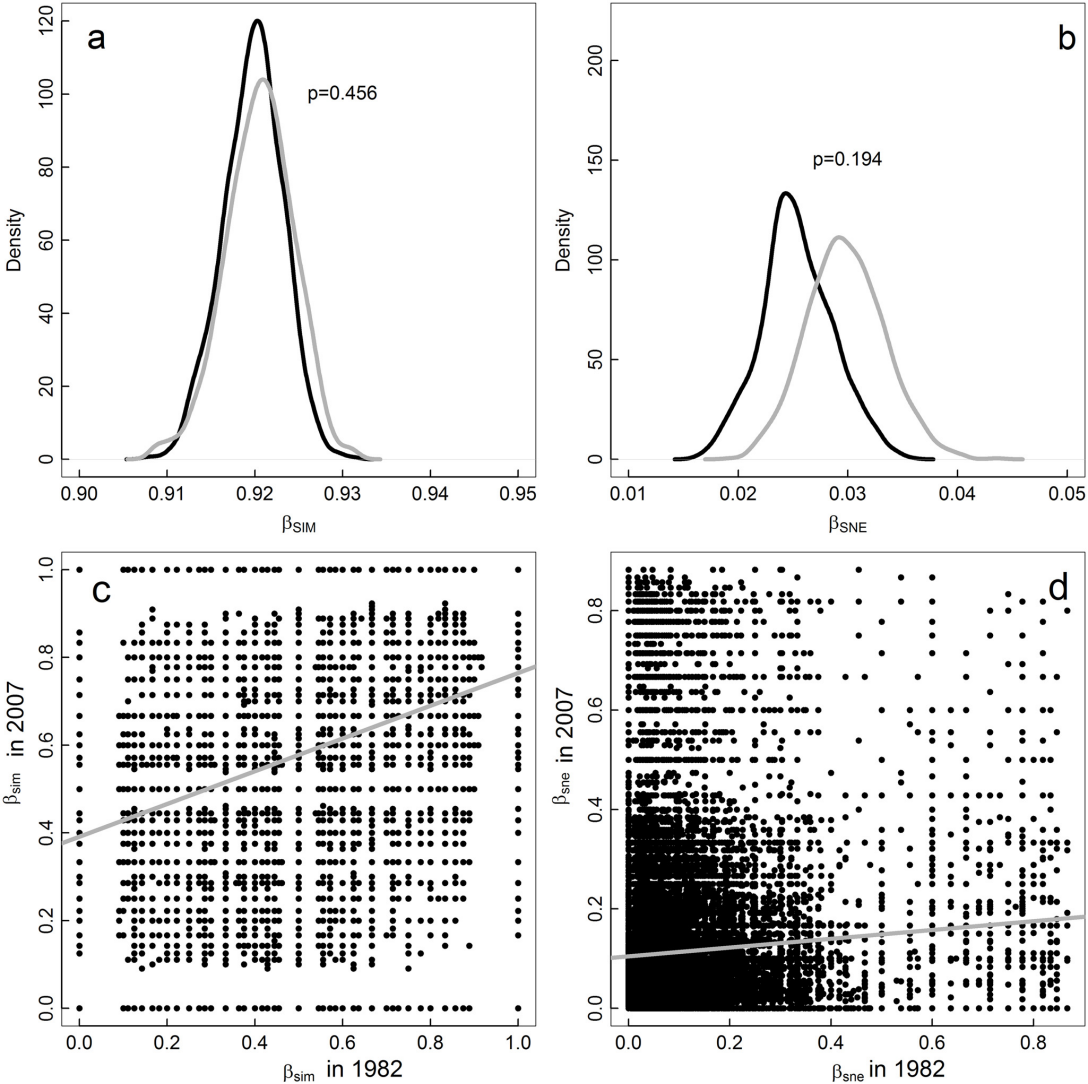
Temporal variation in spatial beta diversity. (a) and (b) show the distribution of multiple site dissimilarity in 1982 (black) and 2007 (gray) for the turnover (β_SIM_) and nestedness-resultant (β_SNE_) components, respectively, after resampling multiple-site dissimilarity values for 50 localities 1000 times. (c) and (d) show the correlation between spatial pairwise dissimilarity in 1982 and spatial pairwise dissimilarity in 2007 for the turnover (β_sim_) and nestedness-resultant (β_sne_) components, respectively. The gray lines are the linear fits.

The amount of spatial assemblage heterogeneity, as measured by the multiple-site dissimilarity in the region, did not significantly change between 1982 and 2007, neither for the turnover (β_SIM_) nor for the nestedness-resultant (β_SNE_) component (Fig 2a and 2b). Although β_SIM_ tended to be higher in 2007 (i.e. the spatial turnover tended to increase), the distributions of resampled values were broadly overlapping. In contrast, the spatial structure of dissimilarities between assemblages completely changed between 1982 and 2007 (Fig 2c and 2d), as evidenced by the low correlations between pairwise spatial dissimilarity matrices for both β_sim_ (r^2^ = 0.12, Mantel p<0.001) and β_sne_ (r^2^<0.01, Mantel p = 0.002). Both results taken together imply that although overall heterogeneity across the region remained similar, the particular dissimilarities between site pairs changed dramatically between 1982 and 2007.

**Fig 3:**
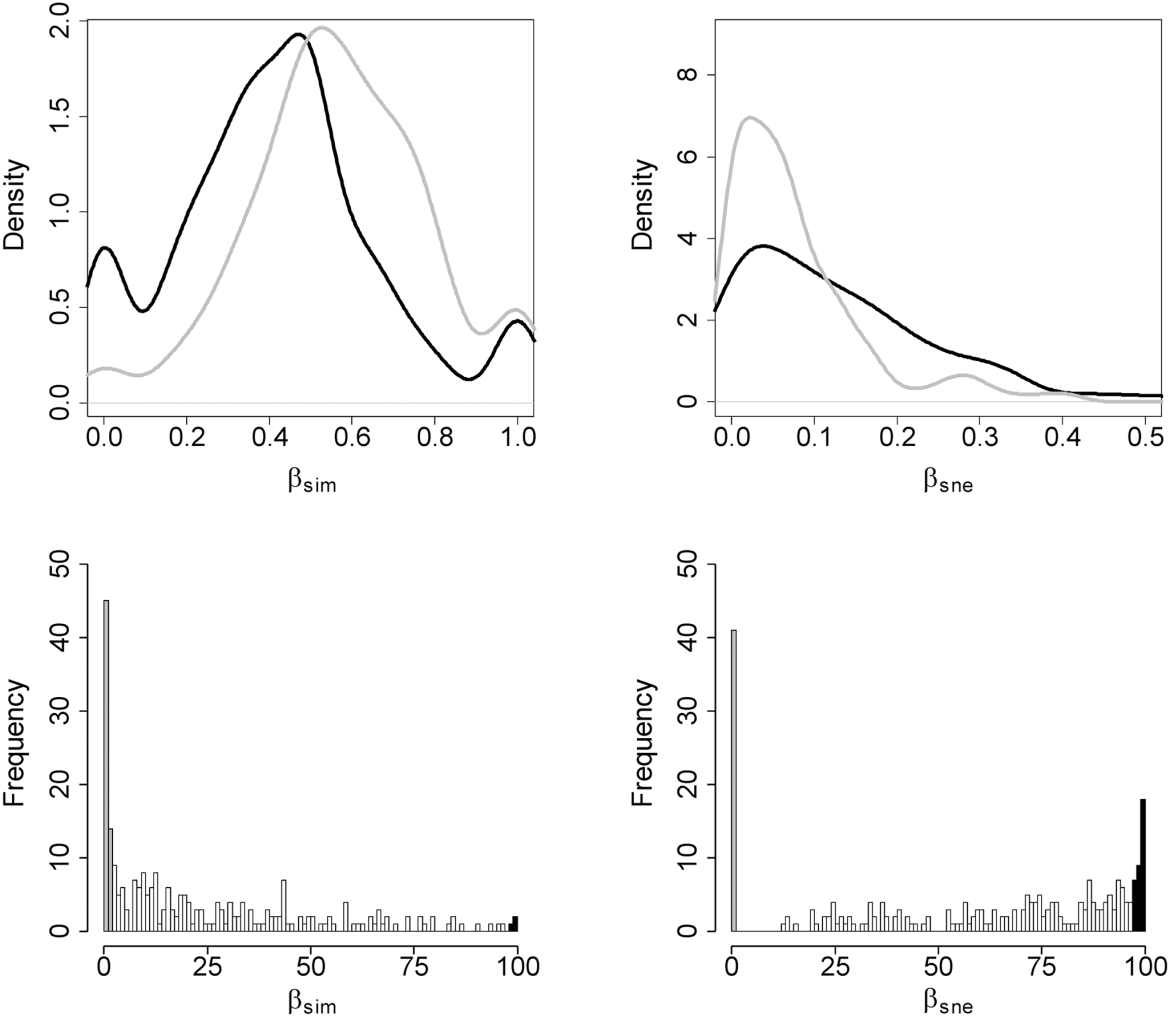
Observed patterns of temporal beta diversity against null expectations. (a) and (b) show the comparison between the distribution of observed temporal dissimilarities (1982 vs. 2007, black) and the distribution of null temporal dissimilarities (1982 vs. null assemblages, gray), for the turnover (β_sim_) and nestedness-resultant (β_sne_) components, respectively. (c) and (d) show the frequency distribution of sites for which the observed temporal turnover is significantly lower (gray) or higher (black) than the null expectation (random reshuffling of the regional pool fixing species frequencies).

The use of a null model reshuffling the regional pool with fixed species frequencies allowed the comparison of observed temporal dissimilarities with the null dissimilarities. The distributions of observed temporal turnover (β_sim_) and nestedness-resultant (β_sne_) turned out to be significantly different from null distributions in all 1000 trials for both β_sim_ and β_sne_ (Kolmogorov-Smirnov D>0.17, p<0.001). Compared to null dissimilarity distributions, observed β_sim_ was biased towards lower values, whereas observed β_sne_ was biased towards higher values, implying that observed temporal species replacement was lower than random reshuffling and temporal nested-resultant dissimilarity patterns were higher than expected. Despite being significantly different, the distributions of observed and null dissimilarities were qualitatively similar (Fig 3a and 3b). When site-specific observed temporal dissimilarities were compared with null expectations, it turned out that in 56 localities (23%) the observed temporal turnover (β_sim_) was significantly lower than expected from the null model, and in only 3 localities (1%) temporal turnover was higher than expected. In the case of nestedness-resultant temporal dissimilarity (β_sne_), 41 localities (16%) presented lower values than expected and 34 localities (13%) presented higher values than expected from the null model (Fig 3c and 3d).

Sites with temporal dissimilarities lower than expected did not show significantly lower values of land cover change, either for β_sim_ and β_sne_ (all r^2^<0.01, F_1,250_<1.67, p>0.19). In turn, localities with higher temporal turnover (β_sim_) than expected showed significantly higher reductions in hedgerow and grassland area, and significantly higher increments in crops; and localities with higher nestedness-resultant dissimilarity than expected showed significantly higher reductions of hedgerow and significantly higher increments of crops. However, the explained variation of these significant relationships was extremely low in all cases (all r^2^<0.04, F_1,250_<9.57, p<0.02).

## Discussion

Our results revealed the existence of (i) moderate temporal turnover, i.e. moderate temporal species substitution rate in local bird assemblages of southwestern France between 1982 and 2007 and (ii) low nestedness-resultant dissimilarity in local assemblages, i.e. small temporal changes related to subset patterns, despite small but significant reductions in local species richness between 1982 and 2007 [18]. The temporal turnover of local assemblage composition (i.e. around 40% of the species in the poorest time were not present in the richest time) was not, or weakly (r^2^<0.06), related to the land cover variables measured in this study. In turn, when compared to a null model simulating the random reshuffling of the regional pool, it turned out that most of these temporal changes in local assemblages were not significantly different from null expectations. Interestingly too, local changes in species composition did not result in changes in overall total multiple-site dissimilarity, which remained relatively constant between 1982 and 2007. In contrast, the particular spatial dissimilarities between site pairs changed dramatically between 1982 and 2007. Taken together, our results suggest that land cover changes occurring in agricultural landscapes of southwestern France between 1982 and 2007 were poor predictors of compositional changes of local bird assemblages. Instead, the observed changes in species composition of local bird assemblages might be the consequence of stochastic processes in which species populations appeared and disappeared from specific localities in a random-like way. A strong stochastic component of temporal assemblage variation has also been recently reported in American birds [6].

Of course, finding patterns compatible with the null hypothesis (in this case stochastic temporal variation in local assemblages) does not imply validating it. We could either (i) have used inappropriate scales and variables to examine changes in assemblage composition, or (ii) have incompletely sampled bird communities, leading to random variation in species composition. The possibility that we have missed the relevant predictors seems unlikely given the land cover factors considered have been repeatedly shown to be relevant predictors of bird species presence and/or abundance [30, 31]. The only possibility we see for having missed relevant drivers would be that processes acting at different scales could be driving local communities. For example, it could be possible that unmeasured environmental changes at larger scales could be affecting demographic parameters and consequently the local assemblages we sampled [32, 33]. However, these processes potentially acting at larger scales did not produce discernible effects on the overall heterogeneity among local assemblages (i.e. multiple site dissimilarity), implying that there is not a consistent pattern modifying the global heterogeneity of the regional assemblage. Another possibility is that the fine scale (i.e. grain) at which we have measured the composition of bird assemblages (5 ha) might not be appropriate for reflecting dynamic processes acting at this temporal scale (25 years) [34–36]. The second problem (incomplete sampling) is unlikely to affect our data, as the sampling protocol has been shown to provide reliable inventories in many previous studies [37]. Thus, we were not able to find any potential mechanistic predictors of temporal variation in assemblage composition and we could not generally reject the null hypothesis of these changes being the result of stochastic processes, which leads to the conclusion that the most likely interpretation is that temporal changes in species composition (i.e. temporal beta diversity) were indeed derived from stochastic assemblage variation. It should be stressed, however, that temporal presence/absence beta diversity is only one aspect of assemblage variation, so our findings do not rule out the presence of deterministic processes on the variation of other assemblage attributes (i.e. species abundances, presence of particular species, among others).

Assuming the major relevance of stochastic processes in temporal beta diversity as a working hypothesis, the interesting question is why temporal changes in species composition of bird assemblages in SW France are weakly related to land cover change. Many reasons may explain this result. A first reason would be that bird species could show time-lagged responses to land-cover changes (higher than our temporal period), with different response shapes according the species traits and thus troubling the response of the community [38, 39]. Another potential explanation would be that bird assemblages in this region are the result of historical human disturbance. Bird species can respond to environmental changes by moving from unsuitable to suitable sites, but they can also present phenotypic adaptation and behavioural changes which allow species to persist in situ [40, 41]. Following changes in human activities, some species can thus adapt and persist in sub optimal habitats [42, 43]. In this sense, a previous study based on the same data has revealed marked differences between both dates in species’ habitat selection [18]. If the magnitude of land cover change in historic periods (i.e. hundreds of years) was comparable to present land cover change (i.e. 1982–2007), conforming a dynamic patchy agricultural landscape, then the regional assemblages would have been previously filtered to cope with these changes and would probably consist in a pool of species using the patchy landscape in a random-like fashion. Patchy landscapes like our study system improve the connectivity and the movement of species between habitats and could consequently buffer impacts of moderate land cover changes [44]. This interpretation is especially likely in communities of highly mobile organisms such as birds (whose populations can move very quickly from patch to patch), and particularly in this biological system, in which bird diversity and composition is mostly driven by forest birds [24]. Moreover, forest bird assemblages have been shown to be little affected by forestry management, as cutting management practices have remained similar for hundreds of years [45].

Another major question that arises is why assemblages seemed to vary randomly from 1982 to 2007. This random variation at the assemblage level implies that individual species disappeared from some places and appeared in some others without any correlation to the measured land cover variables, in agreement with the limited ability of land cover variables to predict species distributions in this system [46]. The reasons for these changes in population occupancy should be further investigated, but lability suggests a highly dynamic and probably non-saturated system in which local populations disperse or disappear due to stochastic processes. Alternatively, stochasticity might be only apparent, resulting from source populations frequently colonizing sink populations located in unsuitable habitats [47].

In conclusion, our analyses showed temporal changes in species composition of local communities were generally compatible with null model predictions. Moreover, our results lend support to the idea of temporal beta diversity of bird assemblages being mostly controlled by stochastic processes, based on (i) the weak relationships between temporal beta diversity and land cover change, (ii) the lack of trend in overall heterogeneity across the region, and (iii) the massive temporal changes in spatial pairwise dissimilarities from one period to the other. If stochastic processes are dominant, the ability of correlative and mechanistic models to predict land cover change effects on species composition and thus implement effective conservation strategies would be compromised. Although these observations might be case-specific (i.e. depending on the land cover change intensity, the study scale or the taxa), in a general context they suggest that stochastic processes may be potentially relevant for explaining temporal changes of biodiversity. Therefore, studies assessing the impacts of global change on biological assemblages should consider the existence of non-deterministic processes in addition to deterministic ones.

## Supporting Information

S1 Dataset Species codes in bird presence/absence data. Codes and scientific names of birds recorded in this study.
(CSV)

S2 Dataset Bird presence/absence data for 1982. Table recording the presence (1) or absence (0) of bird species (columns) in sampling sites (rows) in 1982.
(CSV)

S3 Dataset Bird presence/absence data for 2007. Table recording the presence (1) or absence (0) of bird species (columns) in sampling sites (rows) in 2007.
(CSV)

S4 Dataset Land cover data for 1982. Table recording the spatial position (longitude and latitude) and land cover data (hedgerow length, percentage of woodland area, wooded fallow area, juniper fallow area, permanent grassland area, crop area) of sampling sites (rows) in 1982.
(CSV)

S5 Dataset Land cover data for 2007. Table recording the spatial position (longitude and latitude) and land cover data (hedgerow length, percentage of woodland area, wooded fallow area, juniper fallow area, permanent grassland area, crop area) of sampling sites (rows) in 2007.
(CSV)

S1 Fig Relationship between temporal dissimilarity and land standardized cover change. Plots for the turnover and nestedness-resultant components (y-axes) are shown in the left and right column, respectively. Standardized land cover change (x-axes) was measured for the following variables, by rows: temporal variation in hedgerow length, percentage of woodland area, wooded fallow area, juniper fallow area, permanent grassland area, crop area.
(TIF)
