# Supplementary figures and images for: Temporal Beta Diversity of Bird Assemblages in Agricultural Landscapes: Land Cover Change vs. Stochastic Processes
Source: PLoS One. 2015 May 26;10(5):e0127913. doi: 10.1371/journal.pone.0127913 (PMC4444199; doi:10.1371/journal.pone.0127913)

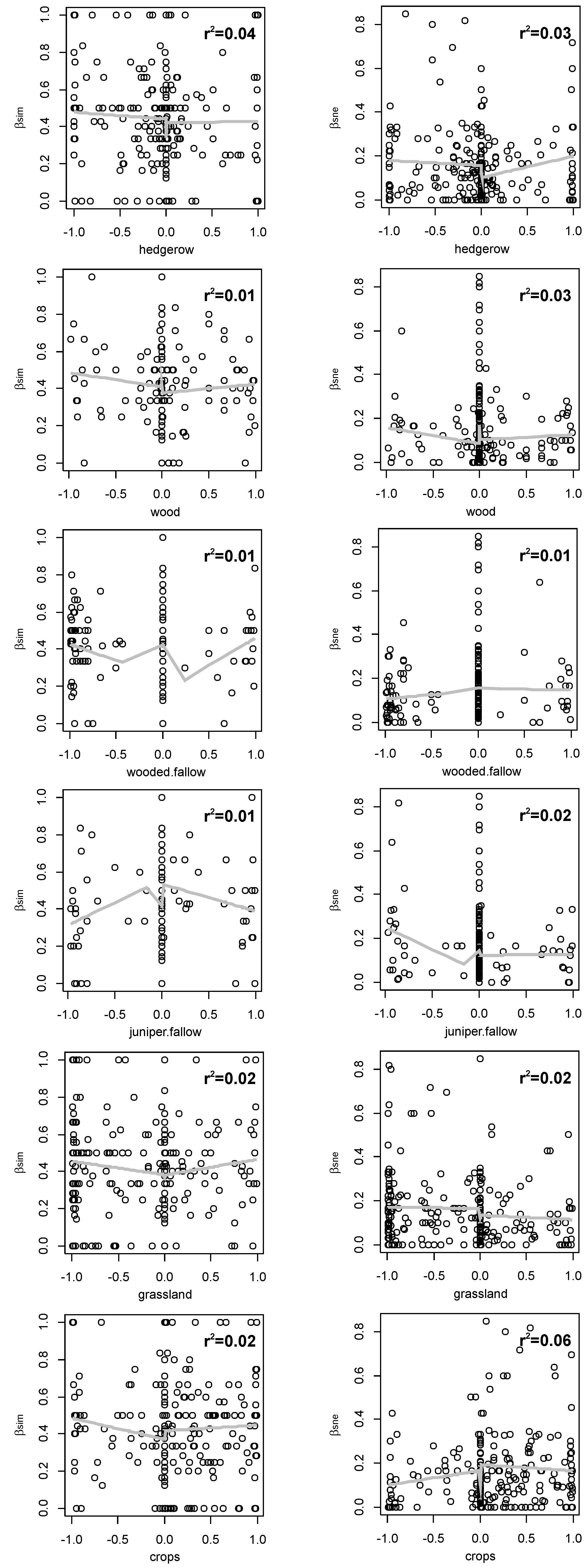

Supplement: S1 Fig — Plots for the turnover and nestedness-resultant components (y-axes) are shown in the left and right column, respectively. Standardized land cover change (x-axes) was measured for the following variables, by rows: temporal variation in hedgerow length, percentage of woodland area, wooded fallow area, juniper fallow area, permanent grassland area, crop area. (TIF) [file pone.0127913.s006.tif]
